# Guidelines for Diagnosis, Treatment, and Follow-Up of Patients with Follicular Lymphoma-Spanish Lymphoma Group (GELTAMO) 2025

**DOI:** 10.3390/cancers18030395

**Published:** 2026-01-27

**Authors:** Francisco-Javier Peñalver, Laura Magnano, Sara Alonso-Álvarez, Ana Jiménez-Ubieto, Armando López-Guillermo, Juan-Manuel Sancho

**Affiliations:** 1 Department of Hematology, Hospital Universitario Fundación Alcorcón, 28922 Alcorcón, Spain; 2 Department of Hematology, Hospital Clínic de Barcelona, 08036 Barcelona, Spain; lcmagnan@clinic.cat (L.M.); alopezg@clinic.cat (A.L.-G.); 3 Department of Hematology, Instituto de Investigación Sanitaria del Principado de Asturias (ISPA)–Hospital Universitario Central de Asturias, 33011 Oviedo, Spain; 4 Department of Hematology, Hospital Universitario 12 de Octubre, 28041 Madrid, Spain; ana.jimenezub@salud.madrid.org; 5 Department of Hematology, ICO-IJC Hospital Germans Trias i Pujol, 08916 Badalona, Spain; jsancho@iconcologia.net

**Keywords:** follicular lymphoma, diagnosis, treatment

## Abstract

Background: Follicular lymphoma (FL) is the second most common B-cell lymphoma in Western countries, typically presenting as an indolent disease with prolonged overall survival. Despite favorable initial responses to therapy, most patients experience relapse, and early progression is associated with poor outcomes. Methods: This guideline provides evidence-based recommendations from the Spanish GELTAMO group on the diagnosis, staging, treatment, and follow-up of FL. A systematic literature review was conducted, and recommendations were graded according to the GRADE system. Results: Histopathological diagnosis should be based on excisional biopsy. PET-CT is recommended for staging and response evaluation. For localized disease, involved-site radiotherapy (ISRT) remains the treatment of choice. In asymptomatic patients with advanced-stage disease and low tumor burden, a watch-and-wait approach is appropriate, although rituximab monotherapy is also acceptable. For advanced-stage disease with high tumor burden, immunochemotherapy with anti-CD20 antibodies (rituximab or obinutuzumab) combined with CHOP, CVP, or bendamustine is recommended, followed by maintenance therapy. Management of relapsed disease is tailored based on tumor burden, treatment history, and timing of relapse. Although novel immunotherapies (CAR-T therapy and bispecific antibodies) are emerging as promising options, autologous stem cell therapies may still be a valid option in young patients with early relapse who are sensitive to immunochemotherapy. Conclusions: FL is a heterogeneous disease requiring individualized management strategies. Recent advances in immunotherapy and molecular diagnostics are reshaping the therapeutic landscape. These updated GELTAMO recommendations aim to provide practical guidance for optimal FL management in clinical practice.

## Simple Summary

Follicular lymphoma (FL) is an indolent B-cell malignancy that typically responds well to first-line therapy. However, many patients experience multiple relapses, and early disease progression is associated with lower survival. These guidelines, developed by the Spanish GELTAMO group, provide an updated, evidence-based overview of the diagnosis, staging, treatment, and follow-up of FL. They summarize current recommendations for optimal clinical management, including the integration of emerging immunotherapies such as CAR-T cell and bispecific antibody therapies. The aim is to support clinicians in personalizing treatment strategies and improving patient outcomes by incorporating the latest therapeutic advances.

## 1. Introduction

Follicular lymphoma (FL) is a B-cell lymphoid neoplasm arising from the cells of the center of the lymphoid follicle in the lymph node and is characterized by a nodular or follicular growth pattern similar to that of the germinal centers [1,2,3]. FL is the second most common type of lymphoma in the Western world, accounting for about 20% of all lymphoma patients, and is considered the paradigm of indolent lymphoma [4]. Although it can occur at any age, FL is more common in older adults (median age at diagnosis, 60 years), and is uncommon in those younger than 20 years. FL patients have a favorable prognosis, with overall survival (OS) reaching or exceeding 20 years from diagnosis according to the most recent studies [5]. However, although patients usually respond well to treatment, most will experience disease relapse.

This guideline, developed by the Spanish Lymphoma Group GELTAMO, provides comprehensive recommendations for the management of FL, encompassing diagnosis, treatment, and follow-up strategies. Formal recommendations and evidence levels are classified using the “GRADE” system [6] which delineates the strength of recommendation (strong—grade 1; weak—grade 2) and the quality of supporting evidence (high—A; moderate—B; low—C; very low—D).

Although these guidelines are grounded in Spanish clinical practice and healthcare organization, its recommendations are primarily based on evidence derived from international clinical trials and consensus statements. Consequently, they may be adapted to different healthcare settings, considering local diagnostic resources, healthcare structures, and drug availability.

## 2. Diagnosis

Excisional biopsy of a lymph node or affected tissue remains the gold standard for diagnosis (1A). Diagnostic strategies must be individualized based on patient frailty, surgical candidacy, disease characteristics, and the accessibility of excisional biopsy. Therefore, Core Needle Biopsy should be reserved for clinically unfit patients or cases with anatomically inaccessible disease [7,8]. Multiple passes with a large-bore needle are required to obtain sufficient material for immunohistochemistry and molecular studies. When histological transformation is suspected, PET/CT-Guided Biopsy is suitable to target the area with the highest metabolic activity [9]. Histological diagnosis should be established according to current recommendations [2,3]. Grade 3B FL is still considered biologically and clinically more similar to diffuse large B-cell lymphoma (DLBCL) than to other forms of FL, and should be treated accordingly.

### 2.1. Histological Classification: WHO (5th Ed) and ICC (2022)

Following the WHO HAEM5 (2022) guidelines, the traditional grading of FL (Grades 1, 2, and 3A) is no longer recommended. These grades are unified under the designation Classic Follicular Lymphoma (cFL), as they share similar clinical behaviors. Individual grading is considered optional. By contrast, grade 3B has been reclassified as a distinct high-grade entity: Follicular Large B-cell Lymphoma (FLBL), which is biologically similar to diffuse large B-cell lymphoma (DLBCL).

The International Consensus Classification (ICC 2022) maintains a similar lineage framework but retains grading (1–2, 3A, 3B) based on centroblast thresholds (>15 centroblasts/HPF for Grade 3), arguing for its continued therapeutic relevance in certain protocols.

### 2.2. Immunohistochemistry and Immunophenotype

FL cells express CD19, CD20, CD22, CD79a, BCL2, BCL6, and CD10. Occasionally, high-grade FL can lose CD10 expression while retaining BCL6 expression. The absence of BCL2 protein expression may be explained by an advanced histological grade or by mutations in *BCL2*.

### 2.3. Cytogenetics and Molecular Biology

FL is characterized by the translocation t(14;18)(q32;q21), which results in rearrangement of the *BCL2* gene with the promoter of the immunoglobulin heavy chain gene or alternatively with light chain genes. Translocation of *BCL2* in isolation is not sufficient for diagnosis of FL. Due to variability in *BCL2* breakpoints, FISH is a very sensitive method for identifying rearrangement and its use is recommended to support diagnosis in cases of atypical histology or variant immunophenotype [1].

Molecular biology techniques can be used experimentally to detect *IGH::BCL2* rearrangement. However, other genetic events can be detected in some FL cases. *BCL6* rearrangement appears in up to 5–15% of FL cases [10], mainly grade FLBL [11]. In certain subtypes (such as the diffuse variant or pediatric FL) [1,12] alterations in 1p36, where the *TNFRSF14* gene is located, are frequent.

While molecular studies are being gradually incorporated into clinical practice, to date their use is not mandatory. The molecular biology of FL follows a hierarchical “trunk and branch” evolutionary pattern. Primary truncal events, include the founding t(14;18) translocation and epigenetic mutations in *KMT2D* and *CREBBP* [13,14]. Meanwhile, secondary events are acquired during progression and/or involve clonal maintenance markers (*TNFRSF14*) [14], immune evasion mechanisms (*B2M*, *CD58*), *MYC* deregulation, biallelic *TP53* inactivation and *CDKN2A/2B* loss [14,15]. This schematic hierarchy underscores the biological complexity of tFL and highlights key drivers of chemoresistance, even where routine genomic profiling remains limited.

Recommendations

• Excisional biopsy of a lymph node or affected tissue is the preferred option for diagnosing FL, provided it is surgically accessible (1A).

## 3. Staging and Prognostic Evaluation

The purpose of the initial evaluation following the diagnosis is to assess parameters relating to tumor extent and burden, as well as other prognostic factors, which will determine treatment. This is achieved through medical history, physical examination, and various additional tests.

### 3.1. Staging

The first step in the staging process is a thorough patient medical history, including assessment for B symptoms (unexplained fever, weight loss, and night sweats). The presence of B symptoms is associated with a higher tumor burden and generally indicates a recommendation for treatment initiation, guided by GELF (*Groupe d’Étude des Lymphomes Folliculaires*), BNLI (British National Lymphoma Society), or SAKK (Swiss Group for Clinical Cancer Research) criteria [16,17,18] (Table 1). The physical examination should meticulously assess all accessible lymph nodes, as well as the spleen and liver. Organomegaly should be documented in centimeters below the costal margin in the midclavicular line, though precise assessment relies on CT findings [19].

Basic laboratory tests are essential for determining parameters relevant to established prognostic indices in FL. These include a complete blood count (with differential count) and serum biochemistry, specifically lactate dehydrogenase (LDH) and beta-2 microglobulin. Protein electrophoresis may identify a monoclonal band in some FL patients. Screening for human immunodeficiency virus (HIV), hepatitis B virus (HBV), and hepatitis C virus (HCV) infections should also be considered. Further laboratory parameters and additional evaluations to assess comorbidities may be beneficial and should be integrated into the comprehensive evaluation of lymphoma patients [19].

PET-CT is the standard imaging modality for FL, used for both initial disease staging and response evaluation. PET-CT offers superior sensitivity compared to conventional CT scans, although contrast-enhanced CT remains valuable in clinical trials for more precise lymphadenopathy measurement. While the “X” suffix for bulky masses is no longer routinely used, documenting the size of the largest mass is recommended. In FL, a bulky mass is typically defined as being between 6 cm [19] and 7 cm [16,18]. Disease staging, consistent with other lymphomas, follows the Lugano classification recommendations [19], which generally categorizes patients into localized (stages I and most stage II) or advanced (stages III and IV) disease for treatment stratification (Table 2). Currently, PET-CT plays an important role in limited-stage FL cases eligible for local radical therapy, both to identify sites not included in the radiotherapy field and in cases with suspected histological transformation, guiding clinicians in choosing the biopsy bases on FDG avidity.

Bone marrow biopsy, with additional immunohistochemical evaluation, remains a necessary component of the initial FL workup [19], and is required in localized stage (1B), but seems to add little value at the time of response evaluation [20,21]. For relapse evaluation, the same initial assessment recommendations apply. Table 3 summarizes the recommended additional tests for FL patients.

Establishing definitive recommendations for routine follow-up imaging to detect early relapse, particularly after achieving metabolic complete remission (CR), remains challenging, similar to other lymphoma subtypes. Retrospective studies [22,23] have shown no significant differences in OS between patients whose relapses were detected via scheduled imaging versus those detected clinically, often by the patients themselves.

### 3.2. Prognostic Evaluation

The prognosis for FL patients is overall favorable, with survival rates now exceeding 20 years, largely attributable to the disease’s indolent nature. Nevertheless, relapses are common and can lead to complications such as histological transformation, which often results in poorer outcomes. FL prognosis is currently determined by factors present at diagnosis, though recent studies increasingly highlight the prognostic significance of certain post-treatment variables. Various prognostic indices based on clinical and biological variables collected at the time of diagnosis have been developed to assess FL outcomes. Information derived from PET-CT and molecular analyses (e.g., gene expression profiling or NGS techniques) can enhance prognostic accuracy. In fact, PET-CT imaging and molecular/NGS-based approaches provide complementary prognostic information in FL PET-CT–based metabolic assessment has been shown to correlate with treatment response and clinical outcomes, whereas molecular analyses—particularly circulating tumor DNA (ctDNA) assessed by ultra-deep sequencing—enable sensitive disease monitoring and early detection of molecular relapse [24,25]. Consequently, new mixed clinical-biological prognostic indices have emerged in recent years, although such molecular testing is not yet routinely integrated into clinical practice [26,27,28]. The tumor microenvironment, particularly the role of immune cells like T lymphocytes and macrophages, is also being studied as a potential prognostic factor; higher T lymphocyte counts generally correlate with improved outcomes. Regarding PET-CT, specific metabolic parameters, such as total metabolic tumor volume (TMTV) and SUVmax, may offer prognostic insights, with proposed cutoff values of 510 cm^3^ [29] and 9.4 g/mL [30], respectively. Despite the increasing number of prognostic scores, their applicability and clinical usefulness remains unclear for several reasons, including the heterogeneity of the therapies, a lack of validation outside front-line treatments, and FL patient survival improvement in recent decades. One of the main limitations of these scores is their limited accuracy in predicting outcomes in individual patients. Currently, these scores are not used when determining the choice treatment [31,32]. Table 4 [26,27,28,33,34,35,36,37] summarizes the primary prognostic scores developed for FL.

Quality and duration of response have recently emerged as critical prognostic factors in FL. PET-CT is a pivotal tool for evaluating treatment response, and persistent disease is clearly associated with a poorer prognosis [38,39]. Similarly, minimal residual disease (MRD), detected using techniques such as polymerase chain reaction (PCR) for *IGH:BCL2* rearrangement, is a significant prognostic factor for progression-free survival (PFS) and OS [24,25]. MRD assessment in FL is an emerging tool, and there is currently no universally accepted standard regarding thresholds, preferred assays, or optimal timing. Although PCR-based MRD assessment remains the current reference method, the FOLL-12 [40] study did not demonstrate that PCR-based MRD assessment can guide or modify treatment decisions. More recent studies using circulating tumor DNA (ctDNA) have shown remarkable sensitivity and strong prognostic value, although evidence that ctDNA-guided MRD monitoring can influence therapeutic decisions is still lacking [24,25,40]. Nonetheless, these advances are promising, and ctDNA-based MRD monitoring may be incorporated into routine clinical practice in the near future.

While most patients respond well to rituximab-based immunochemotherapy, approximately 15–20% do not respond or experience early relapse, defined as progression of disease within 24 months (POD24) from the start of treatment. These patients typically have a significantly worse prognosis, characterized by reduced survival, a higher risk of histological transformation, and a greater probability of treatment failure with rescue therapy. These challenging cases have been a major focus of recent clinical research.

Recommendations

• In addition to standard tests, it is recommended to perform PET-CT for both FL staging and response evaluation (1A).
• Bone marrow biopsy is mandatory to confirm localized FL (1B).
• While prognostic indices (FLIPI, FLIPI2, etc.) can predict outcomes for FL patients, they are not typically useful to guide treatment decisions (1A).
• Patients with FL treated with immunochemotherapy who experience early relapse (e.g., POD24) have an unfavorable prognosis (1A).

## 4. Treatment

### 4.1. First-Line Treatment

**Figure 1:**
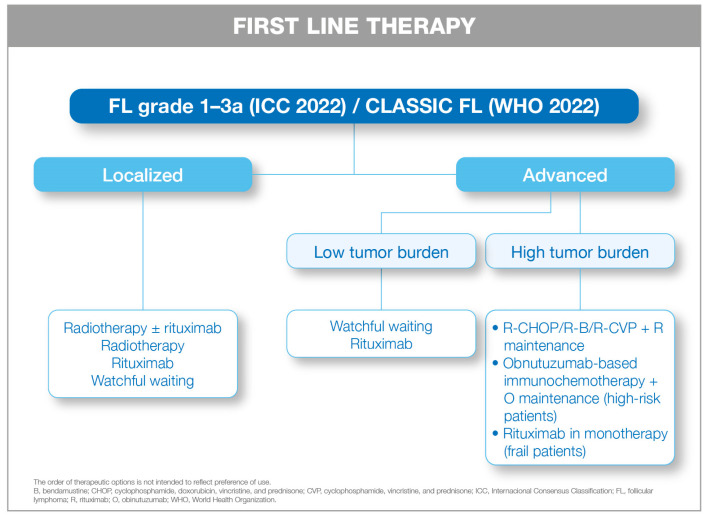
First line therapeutic approach in FLa by disease stage.

The selection of first-line treatment for FL is individualized, depending on factors such as patient age, comorbidities, performance status, tumor burden, disease extent, and patient preferences. At diagnosis, two primary scenarios are distinguished: localized and advanced stages (Figure 1).

#### 4.1.1. Treatment of Localized Stages (Contiguous, Non-Bulky Stages I and II)

Approximately 10% of patients present with localized disease. ISRT at a dose of 24 Gy is the treatment of choice (1A), demonstrating curative potential [41,42,43]. Lower radiotherapy doses (4 Gy) offer less durable efficacy but may be beneficial in specific anatomical locations (e.g., lacrimal gland, parotid glands) to minimize adverse effects (2B) [42,44]. The addition of immunochemotherapy increases PFS but also significantly increases toxicity, and is therefore not recommended [45]. Conversely, the combination of radiotherapy with rituximab (one weekly dose for 4 weeks) provides a favorable balance between efficacy and toxicity (2B) [46]. For patients who are not candidates for radiotherapy due to conditions predisposing to adverse effects (e.g., dry syndrome, hypothyroidism), rituximab monotherapy (1 weekly dose of 375 mg/m^2^ for 4 weeks) may be considered. Patients with bulky disease or other risk factors should be managed as advanced disease. In selected stage I FL patients, radical surgery may represent a potential exclusive treatment option.

#### 4.1.2. Treatment of Advanced Stages [Stages III and IV or Bulky Localized Disease]

Treatment initiation is indicated when the patient is symptomatic or exhibits organ involvement. Two distinct situations are recognized: patients with low tumor burden and those with high tumor burden.

##### Patients with Low Tumor Burden

These patients may be managed with observation, including regular clinical and laboratory monitoring (1A). The median time to requiring treatment is 3 years, and some patients may never require intervention [47]. Routine surveillance imaging is not required unless progression is suspected. Three randomized studies conducted in the pre-rituximab era demonstrated that early chemotherapy treatment initiation in these patients did not improve OS [48,49,50]. In the rituximab era, a single randomized study comparing watchful waiting versus 4 doses of rituximab or 4 doses of rituximab followed by 2 years of rituximab maintenance, showed that early rituximab monotherapy improved PFS but neither OS nor the risk of transformation [51]. Therefore, for this patient group, both watchful waiting and rituximab monotherapy are considered appropriate options (1A). Given the absence of benefit of rituximab maintenance in terms of OS or histological transformation [51,52], the authors recommend 1 weekly dose of 375 mg/m^2^ for 4 weeks for those patients in whom rituximab monotherapy was the chosen option.

##### Patients with High Tumor Burden

- Induction Therapy

Patients with high tumor burden require active treatment. If the primary objective of first-line treatment is to achieve the highest possible CR rates and prolonged PFS, then immunochemotherapy is the treatment of choice (1A). However, no definitive consensus exists regarding the optimal regimen (Table 5). Several immunochemotherapy regimens have been evaluated. R-Bendamustine (R-B) and R-CHOP demonstrate similar efficacy (1B), though R-B is associated with lower toxicity [53], exhibiting reduced rates of neutropenia, mucositis, and absence of alopecia. Nonetheless, for patients with aggressive disease or evidence of transformation, R-CHOP is preferred. Fludarabine-containing combinations have largely fallen out of favor due to significantly higher toxicity compared to R-CHOP or R-B [54,55].

R-CVP is less effective in terms of PFS outcomes but represents a viable option for frail patients [55]. Rituximab monotherapy (at least 1 weekly dose of 375 mg/m^2^ for 4 weeks) has also been utilized in selected high tumor burden patients, offering lower efficacy than immunochemotherapy but with minimal toxicity, making it suitable for cases such as those with significant comorbidities or advanced age (2B) [52,66]. Obinutuzumab, a humanized anti-CD20 type II monoclonal antibody increases the ability to activate NK cells, macrophages and monocytes, achieving greater direct cellular and antibody-mediated cytotoxicity and phagocytosis compared to rituximab. The Phase 3 GALLIUM clinical trial compared rituximab or obinutuzumab combined with chemotherapy regimens (CHOP, CVP, and bendamustine) and followed by rituximab or obinutuzumab maintenance. This trial demonstrated the superiority of obinutuzumab over rituximab in prolonging PFS, particularly in patients with high FLIPI scores, albeit without a significant difference in OS and higher toxicity (1B) [67,68]. Based on these results, obinutuzumab-based immunochemotherapy could be an appropriate option especially for high-risk FLIPI patients (1B). The chemotherapy-free regimen of rituximab and lenalidomide (R^2^) demonstrated similar efficacy than immunochemotherapy (R-CHOP, R-B, and R-CVP) in the Phase 3 RELEVANCE study, but currently lacks EMA approval (2B) [69].

Several ongoing Phase III clinical trials are currently evaluating bispecific antibody–based combinations in the frontline setting including epcoritamab, odronextamab and mosunetuzumab (EPCORE FL-2 [NCT06191744], OLYMPIA-2 [NCT06097364], and MORNINGLYTE [NCT06284122], respectively), which may further expand first-line treatment options for FL in the future.

- Maintenance Therapy

For patients achieving at least a partial response following immunochemotherapy, maintenance with rituximab at 375 mg/m^2^ every 8 weeks for 2 years can significantly prolong PFS (median PFS: 10.5 years vs. 4.1 years; *p* < 0.0001), and has thus become a standard approach for these patients (1A) [40,63]. However, it is important to note that rituximab maintenance has not demonstrated an improvement in OS. Therefore, for patients with recurrent infections, the benefit of this strategy must be carefully weighed. In patients who have received R-B, subsequent rituximab maintenance is more controversial. Recent studies indicate that it also prolongs PFS, particularly in those achieving a partial response (PR) after induction, but without an OS benefit and with increased hematological and infectious toxicity. Consequently, its use should be individualized, considering the toxicity profile and patient preferences [70,71].

Intensification with high-dose chemotherapy followed by autologous stem cell transplantation (ASCT) after first-line treatment has not demonstrated a clear benefit and is therefore not recommended [72].

Recommendations

• In patients with localized FL (contiguous, non-bulky stage I or II), local radiotherapy with curative potential is recommended (1A).
• The addition of rituximab to radiotherapy in patients with localized FL may be considered as a strategy to prolong PFS (2B).
• For advanced stages with low tumor burden, observation is recommended (1A); however, rituximab monotherapy is also a valid option (1A).
• For advanced stages with high tumor burden, immunochemotherapy (R-CHOP, R-CVP, or R-bendamustine) is recommended (1A). Obinutuzumab-based immunochemotherapy may be considered for high-risk patients (1B). Rituximab monotherapy should be reserved for selected cases, such as elderly patients or those with comorbidities (2B).
• In patients who achieve complete response (CR) or partial response (PR) after induction, maintenance with an anti-CD20 antibody (every 2 months for a maximum of 2 years) is recommended (1A), provided that treatment is adequately tolerated.

### 4.2. Treatment of Relapse

If relapse or progression is suspected, a new excisional biopsy is recommended to rule out transformation to a high-grade lymphoma (1A). If feasible, the biopsy should be preferably performed in the area with the highest tracer uptake on the PET-CT scan.

The therapeutic approach to relapse requires a complete re-evaluation to ascertain the tumor burden and disease extent, and thorough assessment of the patient’s clinical status and associated comorbidities (1A). The primary goals are to alleviate associated symptoms, achieve the deepest possible response, and optimize patient quality of life. Treatment selection requires careful weighing of associated risks and benefits, considering factors such as age, comorbidities, tumor burden (or disease stage), associated symptoms, prior treatments and corresponding durations of response, treatment-related toxicities, therapeutic objectives, and patient preferences (1A).

#### 4.2.1. Treatment of Relapse with Low Tumor Burden

For asymptomatic patients with low tumor burden (same criteria than as in first-line; Table 1) management can be similar to first-line therapy. Localized relapse, using PET staging, can be treated with ISRT at a dose of 24 Gy, although a dose of 4 Gy may be a useful alternative for sustained local control and minimized toxicity (2B) [43,46,73]. Disseminated disease with low tumor burden can also be managed with observation or rituximab monotherapy (1B) [74].

#### 4.2.2. Treatment of Relapse with High Tumor Burden

Patients with high tumor burden (Table 1) should receive rescue treatment (1A) (Table 6).

#### 4.2.3. Treatment of Early Relapse (POD24)

Patients experiencing early relapse should be treated if they have a high tumor burden (1B). Given the lack of standard treatment and the reliance on expert opinion, inclusion of these patients in clinical trials is strongly encouraged (1C). Immunochemotherapy, utilizing a regimen different from the first-line regimen, remains the conventional option (1C). The most commonly employed rescue immunochemotherapy regimens include R-CHOP [95] and R-B [60,96]. Generally, R-CHOP can be used as rescue therapy if R-B or rituximab monotherapy was used initially, or if anthracyclines were not part of the first-line treatment [75]. Patients who received first-line R-CHOP or R-CVP can undergo rescue therapy with bendamustine-based regimens [60,96] (1B). The combination of rituximab and lenalidomide (R^2^) can also be considered as a rescue option (1B) [74,77]. Obinutuzumab may be considered in patients refractory to rituximab (1B) [76]. In the absence of data from randomized trials, combination therapy of tafasitamab-R^2^ or bispecific antibodies with lenalidomide or R^2^ could be promising options for this population of patients with early and later relapse. For elderly or frail patients, or those with significant comorbidities, rituximab monotherapy or palliative regimens may be appropriate.

If the patient achieves at least a partial response after induction, additional treatment should be considered to prevent or delay subsequent relapse. In transplant-eligible patients, consolidation with high-dose chemotherapy followed by ASCT can be considered as a therapeutic option, particularly in those with chemosensitive disease (1B) [97,98]. Platinum-based regimens are typically reserved for patients who are transplant candidates. However, the indication for ASCT should be carefully evaluated on an individual basis, considering factors such as age, comorbidities, prior treatments, response to therapy, and the availability of alternative therapeutic approaches. Although randomized studies specifically examining the role of ASCT in relapse in the rituximab era are lacking, existing evidence suggests a potential benefit in selected patients with early relapse or disease refractory to immunochemotherapy, as evidenced by a plateau in PFS curves with prolonged median follow-up. ASCT yields better results when performed at the first or second relapse (1B) [98], with favorable outcomes observed in POD24 patients [99]. Therefore, ASCT may be a consolidation option for eligible POD24 patients who achieve at least a partial response (PR) following rescue therapy [100]. However, this option is becoming less popular while other therapeutic options are available. In patients are not eligible for transplant candidates, maintenance treatment with rituximab may be administered every 3 months for 2 years, provided they have not been previously refractory (1B). However, this strategy is not recommended if relapse occurs during maintenance. Future advancements, particularly the early integration of CAR T-cell therapy, may supersede this management approach.

Recommendations

• Patients with early relapse (POD24) should be treated if they have a high tumor burden (1B).
• A different immunochemotherapy regimen from that used in the first line is recommended (1C) such as R-CHOP, R-B, platinum-based regimens for ASCT candidates, or the combination of rituximab with lenalidomide (R^2^) (1B).
• Obinutuzumab may be considered as an alternative in patients refractory to rituximab (1B).
• Whenever possible, these patients should be considered for inclusion in clinical trials (1C).
• POD24 patients who respond to rescue therapy (achieving at least PR) may benefit from intensification with ASCT, if eligible based on age and general health status (1B).
• For patients not eligible for transplantation, maintenance treatment with rituximab every 3 months for 2 years may be considered (if not previously refractory) (1B).

#### 4.2.4. Treatment of Late First Relapse

There is currently no consensus on the optimal therapeutic option for late first relapse. Immunochemotherapy remains the most frequently used treatment, although published data are limited, and most available information stems from retrospective studies that included patients not previously treated with rituximab. It is recommended to use a regimen different from that used in the first or previous lines, ideally without cross-resistance or with a distinct mechanism of action (1C). Rescue therapy should include an anti-CD20 monoclonal antibody, rituximab or obinutuzumab [76] (1B). Similarly to early relapse, the most commonly used rescue immunochemotherapy regimens are R-CHOP [70] and R-B [71]. Poorer outcomes following CAR T-cell therapy have been reported when bendamustine was used in the 6–9 months prior to apheresis [101]. R^2^ is an increasingly utilized option and may serve as an alternative rescue option with a different mechanism of action if immunochemotherapy was used previously (1B) [74,102]. Patients treated with obinutuzumab and lenalidomide achieved similar outcomes to those who received R^2^ [78]. Immunochemotherapy and lenalidomide combinations have distinct toxicity profiles that must be considered for optimal rescue treatment selection, particularly in older patients or those with comorbidities.

Maintenance treatment with rituximab in relapse prolongs the duration of response in patients who have responded to rescue treatment. Maintenance rituximab therapy is safe and well-tolerated but is associated with a 10% increased risk of infections (1B) [60,95,96]. Therefore, its use should be individualized, taking into account tumor burden at relapse, the induction treatment used, the response and tolerance to it, and the patient’s opinion and preferences.

#### 4.2.5. Treatment of Second or Subsequent Relapses

In these situations, the treatment goals include symptom relief, management of cytopenia (if present), and improving quality of life. The previously described treatment options can be employed, with a recommendation to select a regimen not previously used, preferably with different mechanisms of action and, if possible, without cross-resistance. Thus, options in this setting include a different immunochemotherapy regimen from that used in the first line (1C), R^2^ (1B) in lenalidomide naïve-patients, or rituximab monotherapy, especially in frail patients (2A), followed by maintenance therapy with rituximab in sensitive patients (1B).

Currently, immunotherapy based on bispecific antibodies and CAR T-cell (CART) therapy is revolutionizing the treatment of many B-cell lymphoid malignancies, particularly FL. The bispecific antibodies currently under development for FL exhibit specificity for CD20 on B cells and CD3 on T cells. Three bispecific antibodies—mosunetuzumab, epcoritamab, and odronextamab—are approved by the EMA for third-line treatment of FL, based on the results of three Phase 2 clinical trials [91,93,94,103,104]. Mosunetuzumab is administered intravenously for a fixed duration, while epcoritamab (subcutaneous) and odronextamab are administered until progression. They demonstrate overall response rates (ORRs) of approximately 80% and CR rates of 60–74%. Toxicity profiles are similar to those of CAR T-cell therapy, primarily involving cytokine release syndrome (CRS) and neurotoxicity (ICANS), though with notably lower frequency and severity. Reported response durations exceed 18 months in patients achieving CR, although longer-term follow-up data are still limited. Mosunetuzumab, with the longest follow-up over 3 years, achieves a PFS of 24 months and a median duration of response of 35.9 months (1B) [105].

Three CD19-targeting CAR T-cell products are approved for the treatment of relapsed/refractory (R/R) FL: tisagenlecleucel (tisacel) and lisocabtagene maraleucel (lisocel) from the third line of therapy, and axicabtagene ciloleucel (axicel) from the fourth line, based on results from various Phase 2 clinical trials (1B) (Table 6). All three compounds yielded excellent responses in heavily pretreated patients, including those with early relapses and prior ASCT, with ORRs of 86–95% and CR rates of 68–84%. The most commonly observed toxicities were Grade ≥ 3 CRS (0–6%) and Grade ≥ 3 ICANS (2–15%). Although efficacy data need to be consolidated with longer follow-up, in the ZUMA-5 trial that evaluated axicel with the longest median follow-up, the 5-year PFS exceed 50% [87]. CAR T-cell therapy can be employed as a rescue therapy starting from the second or third relapse, and in patients who demonstrate no response after any line of treatment. Bispecific antibodies could potentially serve as alternatives to CAR T-cell therapy in any of the previously proposed indications and are also viable treatment options for patients who relapse after CAR T-cell therapy. Based on the results of several ongoing clinical trials, immunotherapy with CAR T-cells or bispecific antibodies could be positioned as future first-line and early relapse treatment options. The optimal sequencing of CAR-T therapy vs. bispecific antibodies in relapsed/refractory FL remains a key and unresolved clinical challenge. Recent expert reviews discuss potential sequencing strategies; however, no prospective or comparative studies in FL have established a definitive sequence [105]. While both approaches demonstrate high efficacy in later treatment lines, current sequencing considerations are based on clinical reasoning rather than evidence, incorporating factors such as patient fitness, disease characteristics, prior therapies, toxicity profiles, and logistical aspects. Bispecific antibodies may represent attractive earlier options due to their off-the-shelf availability, whereas CAR-T therapy may be reserved for selected patients with higher-risk or multiply relapsed disease; however, these strategies remain hypothesis-generating.

Tafasitamab, an anti-CD19 monoclonal antibody administered with R^2^ for 12 cycles, achieved a median PFS of 22.4 months in a Phase 3 trial in which tafasitamab-R^2^ was compared with R^2^ (2B) [81]. In a Phase 2 study with a short follow-up duration in which the anti-CD19 antibody-drug conjugate loncastuximab was administered in combination with rituximab, median PFS was not achieved [82].

Second-generation BTK inhibitors may be a treatment option when used in combination. In patients with R/R FL after ≥2 lines of treatment, zanubrutinib plus obinutuzumab until progression was well-tolerated and resulted in a median PFS of 28 months (2B) [79]. In patients with R/R FL, acalabrutinib with R^2^ achieved an overall response rate of 75.9% and a 12-month PFS of 70% (lenalidomide dose, 20 mg) [80].

The oral EZH2 inhibitor tazemetostat resulted in similar responses in patients with both EZH2-mutated and wild-type FL, with a median PFS of approximately 14 months [84,106]. Tazemetostat in combination with R^2^ failed to achieve median PFS, although the trial in question involved a very short follow-up period [107].

Some of these drugs and combinations may be well-positioned to treat patients who have received more effective strategies and have subsequently lost their response, or as a temporary option until a more definitive therapy becomes available. They may also constitute rescue treatment options for second or subsequent relapses in patients not suitable for CAR T-cell therapy or bispecific antibodies. Regarding future directions, while several recurrent molecular alterations in FL—such as mutations in *EZH2*, *CREBBP*, and *KMT2D*—have important biological and prognostic implications, their role in guiding routine clinical decision-making remains limited at present. With the exception of *EZH2* mutations, which may inform the use of targeted therapies in selected clinical settings, most molecular alterations are not yet routinely used to guide treatment selection outside clinical trials [108]. At this stage, genomic insights are primarily of prognostic and research relevance, and their integration into therapeutic decision-making will depend on the results of prospective, biomarker-driven studies.

**Figure 2:**
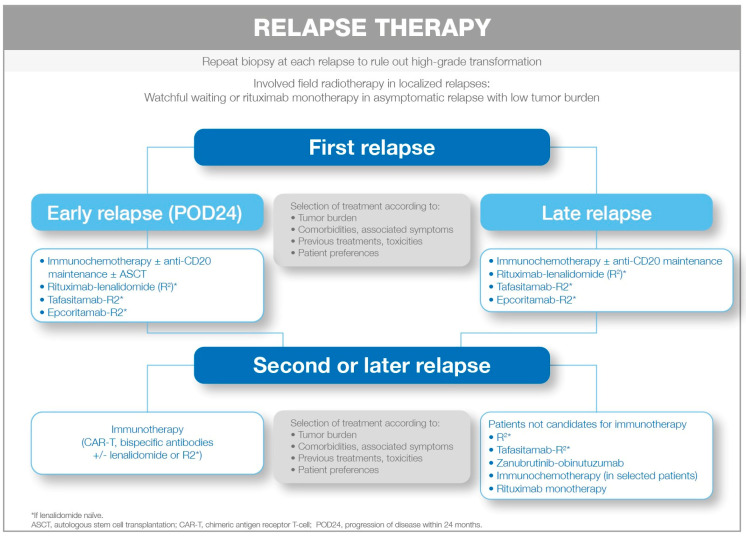
Therapeutic approach for relapsed FL.

ASCT has become less relevant in the setting of subsequent relapses. Allogeneic hematopoietic stem cell transplantation (alloSCT) has demonstrated greater efficacy with a lower number of relapses, but is associated with considerably greater toxicity [109]. Currently, alloSCT (using reduced-intensity conditioning) can be considered in highly selected cases of young patients with good functional status following multiple relapses, typically after treatment with novel agents and CAR T-cell therapy, who have responded to the last rescue therapy. This approach can also be considered for patients without access to CAR T-cell therapy or bispecific antibodies (2C). There are no data on the role of alloSCT as consolidation treatment after CAR T-cell therapy or bispecific antibodies. However, this approach could be considered in refractory patients or those with early relapse after CAR T-cell therapy (Figure 2).

Recommendations

• In cases of suspected relapse or progression, obtaining a new biopsy is recommended to rule out histological transformation (1A).
• Treatment selection for relapse requires an assessment of the risks and benefits of the available options, taking into account age, comorbidities, tumor burden, associated symptoms, duration of response to previous treatment, previous treatments, toxicity to previous treatment and expected toxicity, therapeutic objectives, and patient preferences (1A).
• Localized relapses, without other risk factors, can be treated with involved-site radiotherapy (ISRT) even at low doses (2B).
• Asymptomatic patients with non-localized relapse and low tumor burden can be managed with observation (1B) or rituximab monotherapy (1B).
• For patients treated with induction immunochemotherapy who experience a first relapse/late progression requiring treatment (high tumor burden), the following are recommended:
  ○ Treatment with immunochemotherapy, preferably using a regimen different from that used in the first line (1C), or the combination of rituximab with lenalidomide (R^2^) (1B).
  ○ Rituximab monotherapy (2A).
  ○ Maintenance with rituximab (every 3 months for a maximum 2 years) if at least a partial response is achieved with rescue treatment (1B).
• Second or subsequent relapses can be treated with the following options (preferably if not previously used):
  ○ Immunochemotherapy (1B).
  ○ Rituximab/lenalidomide (R^2^) in lenalidomide naïve-patients (1B).
  ○ Tafasitamab-R^2^ in lenalidomide naïve-patients (2B).
  ○ Zanubrutinib-obinutuzumab (2B).
  ○ Bispecific antibodies: epcoritamab or mosenutuzumab (1B).
  ○ CAR T-cell therapy (tisagenlecleucel in ≥ third line; axicabtagene ciloleucel in ≥ fourth line) (1B).
• Allogeneic transplantation can be considered in young patients with good functional status and without relevant comorbidities, who have relapsed after ASCT, CAR T-cell therapy, and bispecific antibodies, and who have achieved at least a partial response (PR) after rescue treatment (2C).

### 4.3. Management of Transformed Follicular Lymphoma in the Clinical Setting

While anthracycline-based regimens like R-CHOP remain the frontline standard, particularly in anthracycline *naïve* patients, alternative regimens may be preferred in those previously exposed to anthracycline, alternative regimens may be preferred [110]. The clinical utility of Pola-R-CHP in tFL is currently undefined due to the exclusion of transformed histologies from pivotal trials [111]. In the relapsed/refractory setting, the paradigm is shifting from autologous transplant toward CAR T-cell and bispecific antibody therapies. However, therapeutic integration for tFL has not advanced as rapidly as in de novo DLBCL; primarily due to the frequent exclusion or under-representation of tFL patients in large clinical studies, which limits the availability of high-quality evidence and complicates the interpretation of efficacy in this high-risk population.

Recommendations

• R-CHOP is recommended for anthracycline-naïve tFL patients, largely based on extrapolation from de novo DLBCL data (1B).
• Platinum-based regimens would be preferred in patients with prior anthracycline exposure, although evidence is limited to retrospective series (2C).

### 4.4. Special Considerations

Antiviral prophylaxis is recommended for at least 2 weeks and for more than 2 years after the last dose of rituximab in patients with chronic hepatitis B (HBsAg-positive and viral-DNA-negative) as well as carriers (HBcAb-positive, HBsAg-negative and viral-DNA-negative. Patients who are HBsAg-positive and viral-DNA-positive should undergo specific antiviral treatment) (1A) [112].

Bendamustine should be used with caution in patients aged ≥70 years and in those who have respiratory comorbidities and/or are undergoing immunosuppression treatment with other therapies. During bendamustine induction and maintenance, prolonged anti-infective prophylaxis against *Pneumocystis jirovecii* and antiviral therapy should be administered until a CD4 count >200 cells/µL is achieved on two occasions (1B) [67].

Vaccination in accordance with the vaccination schedule is recommended for patients diagnosed with FL (1A), owing to their increased risk of infections. Recommended vaccines include those against influenza, pneumococcus (VNP23), *Haemophilus influenzae* type b (Hib), and meningococcus (1A).

Recommendations

• Patients with chronic hepatitis B (HBsAg-positive and viral-DNA-negative) as well as carriers (HBcAb-positive, HBsAg-negative and viral-DNA-negative) should receive antiviral prophylaxis and load monitoring. Patients with HBsAg-positive and viral-DNA-positive should undergo specific antiviral treatment (1A).
• Following bendamustine administration, anti-infective prophylaxis against *Pneumocystis jirovecii* is recommended according to local guidelines throughout immunosuppressive treatment and until a CD4 count >200 cells/µL is achieved on two occasions (1B).
• Vaccination is recommended for patients diagnosed with FL (1A).

## 5. Future Directions

Treatment of FL will likely evolve towards increasingly personalized and biology-driven strategies. The integration of molecular and genetic profiles, including mutational analyses, gene expression signatures, and microenvironmental markers, will allow for more precise risk stratification and therapeutic tailoring. Monitoring of minimal residual disease may play a key role in tailoring treatment intensity to depth of response. Advances in immunotherapy, including the early incorporation of CAR-T and bispecific antibody therapies, promise to improve the depth and duration of responses, potentially reshaping current therapeutic sequences. Moreover, rational combinations of targeted agents and immunotherapies can potentially help overcome resistance mechanisms while minimizing treatment-related toxicity. Together, these developments move the field closer to achieving durable remission and, ultimately, a functional cure for FL patients.

## 6. Conclusions

FL remains a biologically and clinically heterogeneous disease requiring individualized management strategies. Advances in diagnostic imaging, molecular profiling, and immunotherapy have substantially improved outcomes and are reshaping current standards of care. These updated GELTAMO guidelines aim to provide evidence-based recommendations that integrate these developments into clinical practice to optimize patient management across all stages of disease.

## Figures and Tables

**Table 1:** High burden criteria in FL according to the *Groupe d’Étude des Lymphomes Folliculaires* (GELF), the British National Lymphoma Society (BNLI), and the Swiss Group for Clinical Cancer Research (SAKK).

GELF Criteria	BLNI Criteria	SAKK Criteria
• Presence of B symptoms • Three or more lymph node areas, each with a diameter > 3 cm • Largest lymph node or extranodal involvement ≥ 7 cm • Splenomegaly below the umbilical line • Pleural or peritoneal effusion regardless of cellular content • Compression syndrome • Leukemization (circulating malignant cells ≥ 5 × 10^9^/L) or cytopenia (neutrophils < 1 × 10^9^/L, platelets < 100 × 10^9^/L)	• B symptoms or pruritus • Rapidly progressive generalized disease in the last 3 months • Bone lesions detected by X-ray or scintigraphy that could cause pathological fractures • Infiltration of a vital organ • Renal infiltration (even if renal function is normal) • Macroscopic liver infiltration • Significant bone marrow infiltration causing peripheral blood cytopenia (leukocytes < 3 × 10^9^/L, hemoglobin < 100 g/L, or platelets < 100 × 10^9^/L)	• B symptoms • At least 3 lymph node areas with lymphadenopathy > 3 cm • Bulky disease (>7 cm) • Clinically significant progression of lymphadenopathy, splenomegaly, or other lymphoma lesions (50% increase in size over a period of at least 6 months) • Symptomatic lymphadenopathy or splenomegaly • Hemoglobin < 100 g/L • Platelets < 100 × 10^9^/L (due to bone marrow infiltration or splenomegaly)

**Table 2:** Revised staging system for primary nodal lymphomas (Lugano classification) *.

Stage	Involvement	Extranodal (E) Status
Limited		
I	One node or a group of adjacent nodes	Single extranodal lesions without nodal involvement
II	Two or more nodal groups on the same side of the diaphragm	Stage I or II by nodal extent with limited contiguous extranodal involvement
II bulky **	II as above with “bulky” disease	Not applicable
Advanced		
III	Nodes on both sides of the diaphragm; nodes above the diaphragm with spleen involvement	Not applicable
IV	Additional noncontiguous extralymphatic involvement	Not applicable

* Adapted from Cheson B, et al. [19]. ** In FL, a bulky mass is typically defined as being between 6 cm [19] and 7 cm [16,18].

**Table 3:** Recommendations for additional tests to be performed in FL patients.

Recommended	• Histological diagnosis • Complete blood count and differential leukocyte count • Serum biochemistry with renal function tests, liver function tests, urates, LDH, beta-2 microglobulin, and protein electrophoresis • Complete serologies for HCV, HBV, HIV • Bone marrow aspirate and biopsy • CT and PET or PET-CT
Optional	• Lymphocytic immunophenotype in PB and BM • Quantitative PCR for *IGH::BCL2* in PB/BM • Cytogenetics in tumor tissue and in BM • Echocardiogram or isotopic ventriculography (especially in patients who will receive anthracyclines) • Other imaging tests depending on patient characteristics or lymphoma presentation (e.g., MRI, ultrasound) • Sample and tissue bank for future studies

BM, bone marrow; CT, computed tomography; FL, follicular lymphoma; HBV, hepatitis B virus; HCV, hepatitis C virus; HIV, human immunodeficiency virus; LDH, lactate dehydrogenase; MRI, magnetic resonance imaging; PB, peripheral blood; PCR, polymerase chain reaction; PET, positron emission tomography; PET-CT, positron emission tomography combined with computed tomography.

**Table 4:** Prognostic indices in FL.

Index	Prognostic Factors	Risk Category (Number of Prognostic Factors)	Percentage of Patients	5-Year PFS	5-Year OS
ILI [33]	• Age > 60 y • ESR ≥ 30 • B symptoms > 1 extranodal location • Elevated serum LDH • Male sex	Low (0–1)	64	NR	90%
		Intermediate (2)	23	NR	75%
		High (≥3)	13	NR	38%
FLIPI [34]	• Age ≥ 60 y • Ann Arbor Stage III–IV • Hemoglobin < 120 g/L • Elevated serum LDH • >4 lymph node areas	Low (0)	36	NR	90.6%
		Intermediate (1–2)	37	NR	77.6%
		High (3–5)	27	NR	52.5%
FLIPI2 [35]	• Age > 60 y • Bone marrow infiltration Hemoglobin < 120 g/L • Bone marrow infiltration • Hemoglobin Elevated beta-2 microglobulin • Lymph node mass > 6 cm	Low 0	20	80%	98%
		Intermediate (1–2)	53	51%	88%
		High (3–5)	27	19%	77%
PRIMA-PI [36]	• Elevated beta-2 microglobulin (>3 mg/L) • Bone marrow infiltration	Low–intermediate	65	69–55%	93–93%
		High ^+^	34	37%	81%
m7-FLIPI [26]	• ECOG > 1 • FLIPI high risk • Mutational status of *EZH2*, *ARID1A*, *MEF2B*, *EP300*, *FOXO1*, *CREBBP*, *CARD11*	Low (<0.8) ^++^	72	NR	90%
		High (>0.8) ^++^	28	NR	65%
POD24-PI [27]	• FLIPI high risk • Mutational status of *EZH2*, *EP300*, *FOXO1*,	Low (<0.71)	58	NR	91%
		High (>0.71)	42	NR	71%
23-Gene-expression profiling score [28]	• *VPREB1*, *FOXO1*, *FCRL2*, *TCF4*, *RASSF6*, *GADD45A*, *E2F5*, *USP44*, *SEMA4B*, *EML6*, *DCAF12*, *VCL*, *RGS10*, *TAGAP*, *ORAI2*, *KIAA0040*, *METRNL*, *PRDM15*, *ALDH2*, *SHISA8*	Low (<1.075)	65	73%	NR
		High (>1.075)	35	26%	NR
FLEX [37]	• Male sex • SPD in the highest quartile • Histological grade 3A • >2 extranodal locations • ECOG > 1 • Hemoglobin < 120 g/L • Elevated beta-2 microglobulin • Elevated LDH • NK cell count in PB < 100/L	Low (0–2)	64	86% (3-year PFS)	97% (3-year OS)
		High (3–9)	36	68% (3-year PFS)	87% (3-year OS)

^+^ A beta-2 microglobulin level >3 mg/L classifies patients as high risk. ^++^ To calculate the m7-FLIPI, see https://www.german-lymphoma-alliance.de/Scores.html (Last access: 14 January 2026). ECOG, Eastern Cooperative Oncology Group Performance Status; ESR, erythrocyte sedimentation rate; FLIPI, International Prognostic Index for Follicular Lymphoma; ILI, Italian Lymphoma Intergroup; LDH, lactate dehydrogenase; NR, not reported; OS, overall survival; PB, peripheral blood; PFS, progression free survival; SPD, sum of the product of lymph node diameters; y, years.

**Table 5:** Summary of first-line treatment studies. Data correspond to the arms treated with rituximab.

Treatment	Reference	Trial (Phase)	N Patients	CR/ORR (%)	PFS	AE
R-CVP	Marcus et al. 2008 [56]	3	159	41/81	Median of 2.3 years	Neutropenia G. 3–4 (24%)
R-CHOP	Hiddemann et al. 2005 [57]	2	223	20/96	Median NA at 5 years	Neutropenia G. 3–4 (63%)
R-MCP	Herold et al. 2007 [58]	3	105	50/92	Median NA at 4 years	Neutropenia G. 3–4 (72%) Infections (7%)
R-CHVP + IFN	Bachy et al. 2013 [59]	-	175	67/81	Median of 5.5 years	Neutropenia G. 3–4 (59%)
R-B	Rummel et al. 2013 [53]	3	261	40/93	Median of 6.5 years	-
R-B + maintenance	Rummel et al. 2017 [60]	3	555	33/90	Median NA at 2.8 years	Infections (11%)
R-B	Flinn et al. 2014 and 2019 [61,62]	3	224	31/97	Median NA at 5.4 years	Second neoplasms (19%)
R-CHOP/R-CVP			223	25/91	Median NA at 5.4 years	Second neoplasms (11%)
R-COP	Federico et al. 2013 [54] Luminari et al. 2018. [55]	3	178	67/88	38% at 8 years	Neutropenia G. 3–4 (28%)
R-CHOP			178	73/93	45% at 8 years	Neutropenia G. 3–4 (50%)
R-FCM + R maintenance			178	72/91	39% at 8 years	Neutropenia G. 3–4 (64%)
R-CHOP/R-CVP/R-FM	Bachy et al. 2019 [63]	3	513	ND	35% at 10 years	Neutropenia G. 3–4 (1.6%) Infections (1%)
R-CHOP/R-CVP/R-FM + R maintenance			505		51% at 10 years	Neutropenia G. 3–4 (5.2%) Infections (4.1%)
R-CHOP/R-CVP/R-B + R maintenance	Marcus et al. 2017 [64]	3	601	-/87	73% at 3 years	Neutropenia G. 3–4 (16%)
O-CHOP/O-CVP/O-B + O maintenance			601	-/89	80% at 3 years	Neutropenia G. 3–4 (11%)
R-CHOP/R-B + R maintenance	Morschhauser, et al. 2018 [65]	3	517	53/84	78% at 3 years	Neutropenia G. 3–4 (50%) Cutaneous reaction (7%)
R-lenalidomide + R maintenance			513	48/89	77% at 3 years	Neutropenia G. 3–4 (32%) Cutaneous reaction (1%)

AE, adverse events; CR, complete response; IFN, interferon; G, grade; NA, not achieved; ND, not determined; O, Obinutuzumab; O-B, Obinutuzumab-Bendamustine; O-CHOP, Obinutuzumab-Cyclophosphamide, Hydroxydaunorubicin, Oncovin, Prednisone; O-CVP, Obinutuzumab-Cyclophosphamide, Vincristine, Prednisone; ORR, overall response rate; PFS, progression free survival; R-B, Rituximab-Bendamustine; R-CHOP, Rituximab-Cyclophosphamide, Hydroxydaunorubicin, Oncovin, Prednisone; R-CHVP, Rituximab-Cyclophosphamide, Hydroxydaunorubicin, Vincristine, Prednisone; R-COP, Rituximab-Cyclophosphamide, Oncovin, Prednisone; R-CVP, Rituximab-Cyclophosphamide, Vincristine, Prednisone; R-FCM, Rituximab-Fludarabine, Cyclophosphamide, Mitoxantrone; R-FM, Rituximab-Fludarabine, Mitoxantrone; R-MCP, Rituximab-Mitoxantrone, Chlorambucil, Prednisone.

**Table 6:** Summary of rescue treatment studies.

Treatment	Reference	Trial (Phase)	No. of Patients	No. of Previous Lines	CR/ORR (%)	mPFS (Months)	AE (G.3–4)
R-CHOP	Van Oers, 2006 [75]	3	234	1 (1–2)	29.5/85.1	31.1	Neutropenia G. 3–4 (54.7%)
R-B	Rummel, 2016 [60]	3	58	1 (1–2)	40/82	54.5	Neutropenia G. 3–4 (9%)
GADOLIN (OB)	Sehn 2016 [76]	3	155	1 (1–≥5)	17/70	25.3	Neutropenia G. 3–4 (37.3%)
R^2^ (AUGMENT)	Leonard, 2022 [77]	3b	147	1 (1–≥4)	34/78	27.6	Neutropenia G. 3–4 (50%)
GALEN (O-Lenalidomide)	Morschhauser, 2019 [78]	2	89	1	38/79	65% (2 y)	Neutropenia G. 3–4 (47%)
ROSEWOOD (Zanubrutinib-O)	Zinzani, 2023 [79]	2	145	≥2	39/69	28	Neutropenia G.3–4 (27.4%) Thrombopenia G.3–4 (14%)
Acalabrutinib-R^2^	Strati, 2025 [80]	1b	21		42.9/76.2	70% (1 y)	Neutropenia G.3–4 (37.9%)
Tafasitamab-R^2^	Sehn, 2024 [81]	3	273	1 (1–≥4)	52/83.5	22.4	Neutropenia G.3–4 (39.8%)
Loncastuximab-R	Alderuccio, 2025 [82]	2	36		75/97		Neutropenia G.3–4 (13%)
PolaBR	Flowers, 2024 [83]	2	39	2 (1–5)	69.2/76.9	18.5	Infections G. 3–4 (36.8%) Neutropenia G. 3–4 (31.6%)
PolaBO	Flowers, 2024 [83]	1b/2	26	2 (1–7)	65.4/88.5	40.5	Neutropenia G. 3–4 (30.8%) Infections G. 3–4 (23.7%)
Tazemetostat	Morschhauser, 2020 [84]	2	99	2 (2–43) wt 3 (2–5) mt	50 (wt)/70 (mt)	14.3 (wt) 14.8 (mt)	Neutropenia G. 3–4 (3%)
Tazemetostat-R^2^	Batlevi, 2020 [85]	1b	41		51.2/97.6	84.8 (1 y)	Neutropenia G. 3–4 (34.1%)
Axicel	Jacobson, 2022 [86] Neelapu, 2024 [87]	2	127	3 (1–10)	92/74	40.2	CRS 78% (G3 ≥ 6%) ICANS 56% (G3 ≥ 15%) Hypogamma (15%) Neutropenia G3 ≥ 25%
Tisacel	Fowler, 2022 [88] Dreyling, 2024 [89]	2	98	4 (2–13)	86/68	57.4% (2 y)	CRS 49% (G3 ≥ 0%) ICANS 4% (G3 ≥ 1%) Hypogamma (9%) Neutropenia G3 ≥ 32%
Lisocel	Morschhauser, 2024 [90]	2	107	3 (2–10)	97/94	81% (1 y)	CRS 59% (G3 ≥ 1%) ICANS 15% (G3 ≥ 2%) Hypogamma (5%) Neutropenia G3 ≥ 15%
Mosunetuzumab	Budde, 2022 [91] Sehn, 2025 [92]	2	90	3 (2–4)	80/60	24	CRS 44% (G3 ≥ 2%) ICANS 6% (G3 ≥ 0%) Neutropenia G3 ≥ 26%
Epcoritamab	Linton, 2024 [93]	2	128	3 (2–4)	82/63	15.4	CRS 66% (G3 ≥ 2%) ICANS 6% (G3 ≥ 0%) Neutropenia G3 ≥ 26%
Odronextamab	Kim, 2024 [94]	2	128	3 (12–13)	81/73	20.7	CRS 57% (G3 ≥ 2%) ICANS 2% (G3 ≥0%) Neutropenia G3 ≥ 39.1%

Acalabrutinib-R^2^, Acalabrutinib-Rituximab-Lenalidomide; AE, adverse events; Axicel, Axicabtagene; CRS, cytokine release syndrome; GADOLIN, GA101 (Obinutuzumab) and Dexamethasone; GALEN, GA101 (Obinutuzumab) and Lenalidomide; ICANS, immune effector cell-associated neurotoxicity syndrome; Lisocel, Lisocabtagene Maraleucel; Loncastuximab-R, Loncastuximab-Rituximab; mt, mutated-type; OB, Obinutuzumab; PolaBR, Polatuzumab Vedotin-Bendamustine-Rituximab; PolaBO, Polatuzumab Vedotin-Bendamustine-Obinutuzumab; R-B, Rituximab-Bendamustine; R-CHOP, Rituximab-Cyclophosphamide, Hydroxydaunorubicin, vincristine, Prednisone; R^2^, Rituximab-Lenalidomide; ROSEWOOD, (Zanubrutinib-O), Zanubrutinib-Obinutuzumab; Tazemetostat, EZH2 inhibitor; Tazemetostat-R^2^, EZH2 inhibitor-Rituximab-Lenalidomide; Tisacel, Tisagenlecleuce; wt, wild-type.

## Data Availability

No new data were created or analyzed in this study.
