# Correction: Peñalver et al. Guidelines for Diagnosis, Treatment, and Follow-Up of Patients with Follicular Lymphoma-Spanish Lymphoma Group (GELTAMO) 2026. *Cancers* 2026, *18*, 395

**DOI:** 10.3390/cancers18121950

**Published:** 2026-06-16

**Authors:** Francisco-Javier Peñalver, Laura Magnano, Sara Alonso-Álvarez, Ana Jiménez-Ubieto, Armando López-Guillermo, Juan-Manuel Sancho

**Affiliations:** 1 Department of Hematology, Hospital Universitario Fundación Alcorcón, 28922 Alcorcón, Spain; 2 Department of Hematology, Hospital Clínic de Barcelona, 08036 Barcelona, Spain; lcmagnan@clinic.cat (L.M.); alopezg@clinic.cat (A.L.-G.); 3 Department of Hematology, Instituto de Investigación Sanitaria del Principado de Asturias (ISPA)–Hospital Universitario Central de Asturias, 33011 Oviedo, Spain; 4 Department of Hematology, Hospital Universitario 12 de Octubre, 28041 Madrid, Spain; ana.jimenezub@salud.madrid.org; 5 Department of Hematology, ICO-IJC Hospital Germans Trias i Pujol, 08916 Badalona, Spain; jsancho@iconcologia.net

The authors would like to make the following corrections to this published paper [1].

**Error in Title**

In the original publication, the year in the title was 2025. Due to the updates made, the date in the title should be updated to 2026. The correct legend appears below. Guidelines for Diagnosis, Treatment, and Follow-Up of Patients with Follicular Lymphoma-Spanish Lymphoma Group (GELTAMO) 2026.

**Error in Figure Legend**

In the original publication, there was a mistake in the legend for Figure 1. Fla is misspelled. The correct legend appears below. **Figure 1**. First line therapeutic approach in FL by disease stage.

**Error in Figure**

In the original publication, there was a mistake in Figure 1. Obnutuzumab is misspelled, it should be Obinutuzumab. The updated Figure 1 appears below.

**Error in Table**

In the original publication, there was a mistake in Table 6. Summary of rescue treatment studies. as published. The line item (G.3–4) should be removed from the table header (next to AE). Additionally, the reference date for the Tafasitamab-R^2^ line should be changed to 2026. The adverse events section of the Mosunetuzumab line should also be changed to CRS 1% and ICANS 3%, and a new line (new reference too) should be added at the end (EPCO-R^2^, Falchi 2026 (95), Phase III, number of patients: 488, No of prior lines: 1 [IQR 1–2]), CR/ORR (%) 83/95; mPFS: not reached, AES:CRS 35% (G3 ≥ 0%) ICANS < 1% (G3 ≥ 0%) Neutropenia G3 ≥ 69%.). Moreover, in the previous version from the Axicel row through the Odronextamab row, the data in the column CR/ORR (%) was showing ORR/CR (%). The correct data is now presented in the following table and in the pdf version showing CR/ORR (%) as stated in the column title. The corrected Table 6 appears below.

**Error in Figure**

In the original publication, there was a mistake in Figure 2. Therapeutic approach for relapsed FL. as published. The structure has been changed for better understanding of the content. The corrected Figure 2 appears below.

**Text Correction**

There was an error in the original publication. Due to the publication of new evidence, new information is included in this paragraph and part of it is rewritten to maintain rigor with this evidence. The reference numbers are also modified in this corrected paragraph.

A correction has been made to 4.2.3. Treatment of Early Relapse (POD24), Paragraph Number 1:

Patients experiencing early relapse should be treated if they have a high tumor burden (1B). Given the lack of standard treatment and the reliance on expert opinion, inclusion of these patients in clinical trials is strongly encouraged (1C). Immunochemotherapy, utilizing a regimen different from the first-line regimen, remains one therapeutic option (1C). The most commonly employed rescue immunochemotherapy regimens include R-CHOP [96] and R-B [60,97]. Generally, R-CHOP can be used as rescue therapy if R-B or rituximab monotherapy was used initially, or if anthracyclines were not part of the first-line treatment [75]. Patients who received first-line R-CHOP or R-CVP can undergo rescue therapy with bendamustine-based regimens [60,97] (1B). Poorer outcomes following CAR T-cell therapy have been reported when bendamustine was used in the 6–9 months prior to apheresis [98]. The combination of rituximab and lenalidomide (R^2^) has emerged as an increasingly used rescue therapy (1B) [74,77]. Obinutuzumab may be considered in patients refractory to rituximab (1B) [76]. Recently published evidence from two phase 3 randomized trials indicates that combination therapy with tafasitamab-R^2^ [81], and epcoritamab-R^2^ [95] may be promising treatment options in patients who relapse after at least one line of therapy. Epcoritamab is a bispecific antibody targeting CD20 on B cells and CD3 on T cells, whereas tafasitamab is an anti-CD19 monoclonal antibody. Twelve cycles of either of these two combinations achieved significantly higher response rates and longer PFS compared with R^2^ in FL patients who had received at least one prior line of therapy (including those with POD24). For elderly or frail patients, or those with significant comorbidities, rituximab monotherapy or palliative regimens may be appropriate.

**Text Correction**

There was an error in the original publication. Due to the publication of new evidence, new information is included in this paragraph and part of it is rewritten to maintain rigor with this evidence. The reference numbers are also modified in this corrected paragraph.

A correction has been made to 4.2.3. Treatment of Early Relapse (POD24), Paragraph Number 2:

If the patient achieves at least a partial response after an immunochemoterapy-based induction regimen, additional treatment should be considered to prevent or delay subsequent relapse. In transplant-eligible patients and those with aggressive disease behavior or suspected transformation, consolidation with high-dose chemotherapy followed by ASCT can be considered (1B) [99,100]. Platinum-based regimens are typically reserved for patients who are transplant candidates. However, the indication for ASCT should be carefully evaluated on an individual basis, considering factors such as age, comorbidities, prior treatments, response to therapy, and the availability of alternative therapeutic approaches. Although randomized studies specifically examining the role of ASCT in relapse in the rituximab era are lacking, existing evidence suggests a potential benefit in selected patients with early relapse or disease refractory to immunochemotherapy, as evidenced by a plateau in PFS curves with prolonged median follow-up. ASCT yields better results when performed at the first or second relapse (1B) [100], with favorable outcomes observed in POD24 patients [101]. Therefore, ASCT may be a consolidation option for eligible POD24 patients who achieve at least a partial response (PR) following rescue therapy with immunochemistry [102]. However, this option is becoming less popular as newer therapeutic options become available. In patients not eligible for transplant, maintenance treatment with rituximab may be administered every 3 months for 2 years, provided they have not been previously refractory (1B). However, this strategy is not recommended if relapse occurs during maintenance. Future advancements, particularly the early integration of CAR T-cell therapy, may supersede this management approach.

**Text Correction**

There was an error in the original publication. To maintain consistency of content with the rest of the manuscript structure, the recommendations in this section are removed.

A correction has been made to 4.2.3. Treatment of Early Relapse (POD24), Paragraph Number 3 (Recommendations).

**Text Correction**

There was an error in the original publication. Due to the publication of new evidence, new information is included in this paragraph and part of it is rewritten to maintain rigor with this evidence. The reference numbers are also modified in this corrected paragraph.

A correction has been made to 4.2.4. Treatment of Late First Relapse, Paragraph Number 1:

There is currently no consensus on the optimal therapeutic option for late first relapse. Immunochemotherapy remains an option in these patients, although published data are limited, and most available information stems from retrospective studies that included patients not previously treated with rituximab. A regimen other than that used in first line is recommended, ideally avoiding cross-resistance or with a distinct mechanism of action (1C). Rescue therapy should include an anti-CD20 monoclonal antibody, rituximab or obinutuzumab [76] (1B). Similarly to early relapse, the most commonly used rescue immunochemotherapy regimens are R-CHOP [70] and R-B [71]. R^2^ is an increasingly utilized option with a different mechanism of action if immunochemotherapy was used previously (1B) [74,103]. Patients treated with obinutuzumab and lenalidomide achieved similar outcomes to those who received R^2^ [78]. Immunochemotherapy and lenalidomide combinations have distinct toxicity profiles that must be considered for optimal rescue treatment selection, particularly in older patients or those with comorbidities.

**Text Correction**

There was an error in the original publication. “Rescue treatment” should be changed to a more precise concept. The reference numbers are also modified in this corrected paragraph.

A correction has been made to 4.2.4. Treatment of Late First Relapse, Paragraph Number 2:

Maintenance treatment with rituximab in relapse prolongs the duration of response in patients who have responded to salvage immunochemotherapy. Maintenance rituximab therapy is safe and well-tolerated but is associated with a 10% increased risk of infections (1B) [60,96,97]. Therefore, its use should be individualized, taking into account tumor burden at relapse, the induction treatment used, the response and tolerance to it, and the patient’s opinion and preferences.

**Text Correction**

There was an error in the original publication. Due to the publication of new evidence, new information is included in a new paragraph.

A correction has been made to 4.2.4. Treatment of Late First Relapse, Paragraph Number 3 (new paragraph):

Combination therapy of tafasitamab-R^2^ [81], and epcoritamab-R^2^ [95] administered for a fixed duration of 12 months, are promising treatment options, if available.

**Text Correction**

There was an error in the original publication. Due to the publication of new evidence, new information is included in this paragraph and part of it is rewritten to maintain rigor with this evidence. The reference numbers are also modified in this corrected paragraph.

A correction has been made to 4.2.5. Treatment of Second or Subsequent Relapses, Paragraph Number 1:

In these situations, the treatment goals include symptom relief, management of cytopenia (if present), and improving quality of life. The previously described treatment options can be employed, with a recommendation to select a regimen not previously used, preferably with different mechanisms of action and, if possible, without cross-resistance. Thus, options in this setting include a different immunochemotherapy regimen (1C), R^2^ (1B) tafasitamab-R^2^ (1A) [81] and epcoritamab-R^2^ (1A) [95], in lenalidomide naïve-patients. Rituximab monotherapy, may also be an option, especially in frail patients (2A). For patients treated with immunochemotherapy, maintenance therapy with rituximab every 3 months for 2 years may be considered in patients who achieve CR or PR (1B). However, with the introduction of newer drugs with different mechanisms of action, any of these therapeutic options are less frequently used.

**Text Correction**

There was an error in the original publication. The reference number used is modified because new references are included in previous paragraphs.

A correction has been made to 4.2.5. Treatment of Second or Subsequent Relapses, Paragraph Number 2:

Currently, immunotherapy based on bispecific antibodies and CAR T-cell therapy is revolutionizing the treatment of many B-cell lymphoid malignancies, particularly FL. The bispecific antibodies currently under development for FL exhibit specificity for CD20 on B cells and CD3 on T cells. Three bispecific antibodies—mosunetuzumab, epcoritamab, and odronextamab—are approved by the EMA for third-line treatment of FL, based on the results of three Phase 2 clinical trials [91,93,94,104,105]. Mosunetuzumab is administered intravenously for a fixed duration, while epcoritamab (subcutaneous) and odronextamab are administered until progression. They demonstrate overall response rates (ORRs) of approximately 80% and CR rates of 60–74%. Toxicity profiles are similar to those of CAR T-cell therapy, primarily involving cytokine release syndrome (CRS) and neurotoxicity (ICANS), though with notably lower frequency and severity. Reported response durations exceed 18 months in patients achieving CR, although longer-term follow-up data are still limited. Mosunetuzumab, with the longest follow-up over 3 years, achieves a PFS of 24 months and a median duration of response of 35.9 months (1B) [106].

**Text Correction**

There was an error in the original publication. Due to the publication of new evidence, new information is included in this paragraph and part of it is rewritten to maintain rigor with this evidence. The reference numbers are also modified in this corrected paragraph.

A correction has been made to 4.2.5. Treatment of Second or Subsequent Relapses, Paragraph Number 3:

Three CD19-targeting CAR T-cell products are approved by EMA for the treatment of relapsed/refractory (R/R) FL: tisagenlecleucel (tisacel) and lisocabtagene maraleucel (lisocel) from the third line of therapy, and axicabtagene ciloleucel (axicel) from the fourth line (approved by FDA in third line), based on results from various Phase 2 clinical trials (1B) (Table 6). All three compounds yielded excellent responses in heavily pretreated patients, including those with early relapses and prior ASCT, with ORRs of 86–95% and CR rates of 68–84%. The most commonly observed toxicities were Grade ≥ 3 CRS (0–6%) and Grade ≥ 3 ICANS (2–15%). Although efficacy data need to be consolidated with longer follow-up, in the ZUMA-5 trial that evaluated axicel with the longest median follow-up, the 5-year PFS exceed 50% [87]. CAR T-cell therapy can be employed as a rescue therapy starting from the second or third relapse, and in patients who demonstrate no response after any line of treatment. Bispecific antibodies could potentially serve as alternatives to CAR T-cell therapy in any of the previously proposed indications and are also viable treatment options for patients who relapse after CAR T-cell therapy. The optimal sequencing of CAR-T therapy vs. bispecific antibodies in relapsed/refractory FL remains a key and unresolved clinical challenge. Recent expert reviews discuss potential sequencing strategies; however, no prospective or comparative studies in FL have established a definitive sequence [106]. While both approaches demonstrate high efficacy in later treatment lines, current sequencing considerations are based on clinical reasoning rather than evidence, incorporating factors such as patient fitness, disease characteristics, prior therapies, toxicity profiles, and logistical aspects. Bispecific antibodies may represent attractive earlier options due to their off-the-shelf availability, whereas CAR-T therapy may be reserved for selected patients with higher-risk or multiply relapsed disease; however, these strategies remain hypothesis-generating.

**Text Correction**

There was an error in the original publication. Part of the paragraph should be removed due to new evidence, and the second part of the paragraph (not removed) should be rewritten in a more precise way for better understanding.

A correction has been made to 4.2.5. Treatment of Second or Subsequent Relapses, Paragraph Number 4:

In a Phase 2 study with a short follow-up duration, the anti-CD19 antibody-drug conjugate loncastuximab administered in combination with rituximab, median PFS was not reached [82], suggesting its potential role as salvage therapy.

**Text Correction**

There was an error in the original publication. The wording of zanubrutinib plus obinutuzumab should be changed.

A correction has been made to 4.2.5. Treatment of Second or Subsequent Relapses, Paragraph Number 5:

Second-generation BTK inhibitors may be a treatment option when used in combination. In patients with R/R FL after ≥2 lines of treatment, obinutuzumab plus zanubrutinib until progression was well-tolerated and resulted in a median PFS of 28 months (2B) [79]. In patients with R/R FL, acalabrutinib with R^2^ achieved an overall response rate of 75.9% and a 12-month PFS of 70% (lenalidomide dose, 20 mg) [80].

**Text Correction**

There was an error in the original publication. It should be rewritten for better understanding. The reference numbers are also modified in this corrected paragraph.

A correction has been made to 4.2.5. Treatment of Second or Subsequent Relapses, Paragraph Number 6:

The oral EZH2 inhibitor tazemetostat resulted in similar responses in patients with both EZH2-mutated and wild-type FL, with a median PFS of approximately 14 months [84,107]. At the time of analysis, tazametostat in combination with R^2^ had not achieved median PFS, although the trial in question involved a very short follow-up period [108].

**Text Correction**

There was an error in the original publication. The reference number used is modified because new references are included in previous paragraphs.

A correction has been made to 4.2.5. Treatment of Second or Subsequent Relapses, Paragraph Number 7:

Some of these drugs and combinations may be well-positioned to treat patients who have received more effective strategies and have subsequently lost their response, or as a temporary option until a more definitive therapy becomes available. They may also constitute rescue treatment options for second or subsequent relapses in patients not suitable for CAR T-cell therapy or bispecific antibodies. Regarding future directions, while several recurrent molecular alterations in FL—such as mutations in *EZH2*, *CREBBP*, and *KMT2D*—have important biological and prognostic implications, their role in guiding routine clinical decision-making remains limited at present. With the exception of *EZH2* mutations, which may inform the use of targeted therapies in selected clinical settings, most molecular alterations are not yet routinely used to guide treatment selection outside clinical trials [109]. At this stage, genomic insights are primarily of prognostic and research relevance, and their integration into therapeutic decision-making will depend on the results of prospective, biomarker-driven studies.

**Text Correction**

There was an error in the original publication. The reference number used is modified because new references are included in previous paragraphs.

A correction has been made to 4.2.5. Treatment of Second or Subsequent Relapses, Paragraph Number 8:

ASCT has become less relevant in the setting of subsequent relapses. Allogeneic hematopoietic stem cell transplantation (alloSCT) has demonstrated greater efficacy with a lower number of relapses, but is associated with considerably greater toxicity [110]. Currently, alloSCT (using reduced-intensity conditioning) can be considered in highly selected cases of young patients with good functional status following multiple relapses, typically after treatment with novel agents and CAR T-cell therapy, who have responded to the last rescue therapy. This approach can also be considered for patients without access to CAR T-cell therapy or bispecific antibodies (2C). There are no data on the role of alloSCT as consolidation treatment after CAR T-cell therapy or bispecific antibodies. However, this approach could be considered in refractory patients or those with early relapse after CAR T-cell therapy (Figure 2).

**Text Correction**

There was an error in the original publication. Due to new evidence and the restructuring of the previous content (we removed recommendations from a previous section), the recommendations must be updated.

A correction has been made to 4.2.5. Treatment of Second or Subsequent Relapses, Paragraph Number 9 (recommendations):

- In cases of suspected relapse or progression, obtaining a new biopsy is recommended to rule out histological transformation (1A).
- Treatment selection at relapse requires a careful assessment of the risks and benefits of the available options, taking into account age, comorbidities, tumor burden, associated symptoms, duration of response to previous treatment, previous treatments, toxicity to previous treatment and expected toxicity, therapeutic objectives, and patient preferences (1A).
- Localized relapses, without other risk factors, can be treated with involved-site radiotherapy (ISRT) even at low doses (2B).
- Asymptomatic patients with non-localized relapse and low tumor burden can be managed with observation (1B) or rituximab monotherapy (1B).
- Patients with early or late first symptomatic POD (first relapse ≤ 24 and > 24 months after of first-line treatment), the following salvage options may be considered:
  ○ A different immunochemotherapy regimen from that used in the first-line treatment (1C).
  ○ Obinutuzumab-bendamustine in rituximab-refractory patients (1B).
  ○ Patients with POD24 who respond to rescue therapy (achieving at least PR) may benefit from treatment intensification with ASCT, if eligible based on age and general health status (1B). For patients with POD24 who are not eligible for transplantation, as well as those with late first POD, maintenance treatment with rituximab every 3 months for 2 years may be considered (if not previously refractory) (1B). Obinutuzumab maintenance following Obinutuzumab-bendamustine may also be considered (1B).
  ○ Rituximab-lenalidomide (R^2^) in lenalidomide naive-patients (1B).
  ○ Epcoritamab with rituximab-lenalidomide (R^2^) in lenalidomide-naïve patients (1A).
  ○ Tafasitamab with rituximab-lenalidomide (R^2^) in lenalidomide-naïve patients (1A).
  ○ Rituximab monotherapy (2A) for frail patients.
  ○ Whenever possible, these patients should be considered for inclusion in clinical trials (1C).

- Patients with second or subsequent relapses can be treated with the following options (preferably agents not previously used, if available):
  ○ Rituximab-lenalidomide (R^2^) in lenalidomide naive-patients (1B).
  ○ Tafasitamab with rituximab-lenalidomide (R^2^) in lenalidomide-naïve patients (1A).
  ○ Epcoritamab with rituximab-lenalidomide (R^2^) in lenalidomide-naïve patients (1A).
  ○ Bispecific antibodies in monotherapy: epcoritamab, mosenutuzumab or odronextamab (1B).
  ○ Zanubrutinib-obinutuzumab (2B).
  ○ CAR T-cell therapy (1B).
  ○ Non-cross-resistant immunochemotherapy (in selected patients) (1C).
  ○ Rituximab monotherapy (2A).
  ○ Allogeneic stem cell transplantation (using reduced-intensity conditioning) can be considered in highly selected cases of young patients with good functional status following multiple relapses, typically after treatment with novel agents and CAR T-cell therapy, who have responded to the last rescue therapy (2C).

**Text Correction**

There was an error in the original publication. The reference number used is modified because new references are included in previous paragraphs.

A correction has been made to 4.3. Management of Transformed Follicular Lymphoma in the Clinical Setting, Paragraph Number 1:

While anthracycline-based regimens like R-CHOP remain the frontline standard, particularly in anthracycline *naive* patients, alternative regimens may be preferred in those previously exposed to anthracycline [111]. The clinical utility of Pola-R-CHP in tFL is currently undefined due to the exclusion of transformed histologies from pivotal trials [112]. In the relapsed/refractory setting, the paradigm is shifting from autologous transplant toward CAR T-cell and bispecific antibody therapies. However, therapeutic integration for tFL has not advanced as rapidly as in de novo DLBCL; primarily due to the frequent exclusion or under-representation of tFL patients in large clinical studies, which limits the availability of high-quality evidence and complicates the interpretation of efficacy in this high-risk population.

**Text Correction**

There was an error in the original publication. The reference number used is modified because new references are included in previous paragraphs.

A correction has been made to 4.4. Special Considerations, Paragraph Number 1:

Antiviral prophylaxis is recommended from at least 2 weeks before starting anti-lymphoma treatment and for more than 2 years after the last dose of rituximab in patients with chronic hepatitis B (HBsAg-positive and viral-DNA-negative) as well as carriers (HBcAb-positive, HBsAg-negative and viral-DNA-negative. Patients who are HBsAg-positive and viral-DNA-positive should undergo specific antiviral treatment) (1A) [113].

**References Correction**

Reference 81 should be replaced by:

81. Sehn, L.H.; Hübel, K.; Luminari, S.; Scholz, C.W.; Salar, A.; Paneesha, S.; Wahlin, B.E.; Panayiotidis, P.; Lee, H.P.; Jiménez-Ubieto, A.; et al. Tafasitamab, lenalidomide, and rituximab in relapsed or refractory follicular lymphoma (inMIND): A global, phase 3, randomised controlled trial. *Lancet* **2026**, *407*, 133–146. https://doi.org/10.1016/S0140-6736(25)01778-7.

A new reference is included in number 95, so the order of the remaining references must be modified:

95. Falchi, L.; Nijland, M.; Huang, H.; Linton, K.M.; Seymour, J.F.; Tao, R.; Kwiatek, M.; Costa, A.; Vassilakopoulos, T.P.; Greil, R.; et al. Epcoritamab, lenalidomide, and rituximab versus lenalidomide and rituximab for relapsed or refractory follicular lymphoma (EPCORE FL-1): A global, open-label, randomised, phase 3 trial. *Lancet* **2026**, *407*, 161–173. https://doi.org/10.1016/S0140-6736(25)02360-8.

A new reference is included in number 98, so the order of the remaining references must be modified:

98. Iacoboni, G.; Navarro, V.; Martin-Lopez, A.A.; Rejeski, K.; Kwon, M.; Jalowiec, K.A.; Amat, P.; Reguera-Ortega, J.L.; Gallur, L.; Blumenberg, V.; et al. Recent Bendamustine Treatment Before Apheresis Has a Negative Impact on Outcomes in Patients with Large B-Cell Lymphoma Receiving Chimeric Antigen Receptor T-Cell Therapy. *J. Clin. Oncol.* **2024**, *42*, 205–217.

Reference 101 (Iacoboni, G.; Navarro, V.; Martin-Lopez, A.A.; Rejeski, K.; Kwon, M.; Jalowiec, K.A.; Amat, P.; Reguera-Ortega, J.L.; Gallur, L.; Blumenberg, V.; et al. Recent Bendamustine Treatment Before Apheresis Has a Negative Impact on Outcomes in Patients with Large B-Cell Lymphoma Receiving Chimeric Antigen Receptor T-Cell Therapy. *J. Clin. Oncol.* **2024**, *42*, 205–217.) must be removed, so the remaining references must be reordered accordingly.

With this correction, the order of some references has been adjusted accordingly. The authors state that the scientific conclusions are unaffected. This correction was approved by the Academic Editor. The original publication has also been updated.

## Figures and Tables

**Figure 1:**
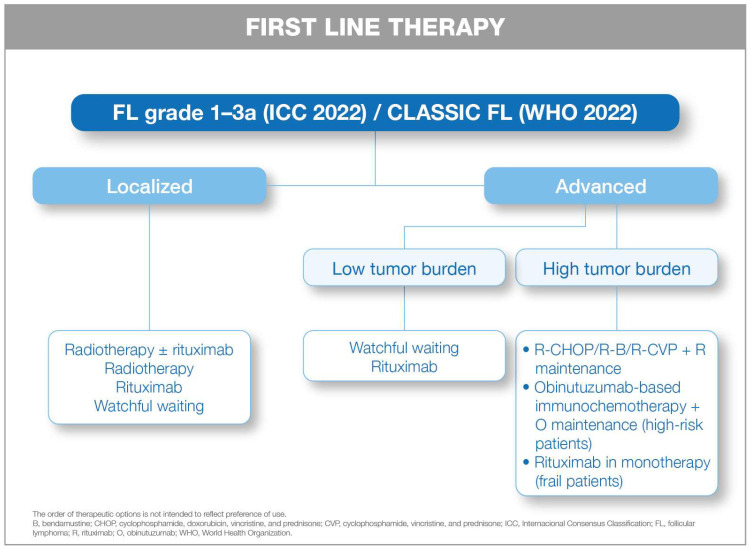
First line therapeutic approach in FL by disease stage.

**Figure 2:**
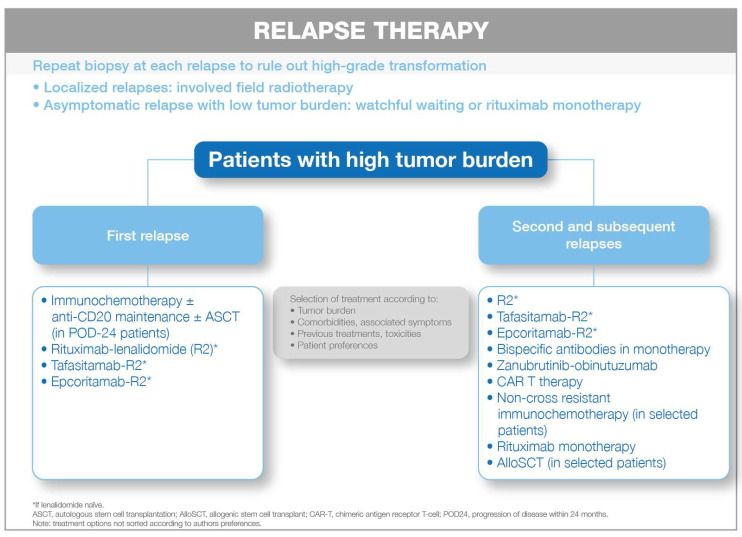
Therapeutic approach for relapsed FL. * If lenalidomide naïve.

**Table 6:** Summary of rescue treatment studies.

Treatment	Reference	Trial (Phase)	No. of Patients	No. of Previous Lines	CR/ORR (%)	mPFS (Months)	AE
R-CHOP	Van Oers, 2006 [75]	3	234	1 (1–2)	29.5/85.1	31.1	Neutropenia G.3–4 (54.7%)
R-B	Rummel, 2016 [60]	3	58	1 (1–2)	40/82	54.5	Neutropenia G.3–4 (9%)
GADOLIN (OB)	Sehn 2016 [76]	3	155	1 (1–≥5)	17/70	25.3	Neutropenia G.3–4 (37.3%)
R^2^ (AUGMENT)	Leonard, 2022 [77]	3b	147	1 (1–≥4)	34/78	27.6	Neutropenia G.3–4 (50%)
GALEN (O-Lenalidomide)	Morschhauser, 2019 [78]	2	89	1	38/79	65% (2 y)	Neutropenia G.3–4 (47%)
ROSEWOOD (Zanubrutinib-O)	Zinzani, 2023 [79]	2	145	≥2	39/69	28	Neutropenia G.3–4 (27.4%) Thrombopenia G.3–4 (14%)
Acalabrutinib-R^2^	Strati, 2025 [80]	1b	21		42.9/76.2	70% (1 y)	Neutropenia G.3–4 (37.9%)
Tafasitamab-R^2^	Sehn, 2026 [81]	3	273	1 (1–≥4)	52/83.5	22.4	Neutropenia G.3–4 (39.8%)
Loncastuximab-R	Alderuccio, 2025 [82]	2	36		75/97		Neutropenia G.3–4 (13%)
PolaBR	Flowers, 2024 [83]	2	39	2 (1–5)	69.2/76.9	18.5	Infections G.3–4 (36.8%) Neutropenia G.3–4 (31.6%)
PolaBO	Flowers, 2024 [83]	1b/2	26	2 (1–7)	65.4/88.5	40.5	Neutropenia G.3–4 (30.8%) Infections G.3–4 (23.7%)
Tazemetostat	Morschhauser, 2020 [84]	2	99	2 (2–43) wt 3 (2–5) mt	50 (wt)/ 70 (mt)	14.3 (wt)/ 14.8 (mt)	Neutropenia G.3–4 (3%)
Tazemetostat-R^2^	Batlevi, 2020 [85]	1b	41		51.2/97.6	84.8 (1 y)	Neutropenia G.3–4 (34.1%)
Axicel	Jacobson, 2022 [86] Neelapu, 2024 [87]	2	127	3 (1–10)	74/92	40.2	CRS 78% (G.3 ≥ 6%) ICANS 56% (G.3 ≥ 15%) Hypogamma (15%) Neutropenia G.3 ≥ 25%
Tisacel	Fowler, 2022 [88] Dreyling, 2024 [89]	2	98	4 (2–13)	68/86	57.4% (2 y)	CRS 49% (G.3 ≥ 0%) ICANS 4% (G.3 ≥ 1%) Hypogamma (9%) Neutropenia G.3 ≥ 32%
Lisocel	Morschhauser, 2024 [90]	2	107	3 (2–10)	94/97	81% (1 y)	CRS 59% (G.3 ≥ 1%) ICANS 15% (G.3 ≥ 2%) Hypogamma (5%) Neutropenia G.3 ≥ 15%
Mosunetuzumab	Budde, 2022 [91] Sehn, 2025 [92]	2	90	3 (2–4)	60/80	24	CRS 1% (G.3 ≥ 2%) ICANS 3% (G.3 ≥ 0%) Neutropenia G.3 ≥ 26%
Epcoritamab	Linton, 2024 [93]	2	128	3 (2–4)	63/82	15.4	CRS 66% (G.3 ≥ 2%) ICANS 6% (G.3 ≥ 0%) Neutropenia G.3 ≥ 26%
Odronextamab	Kim, 2024 [94]	2	128	3 (12–13)	73/81	20.7	CRS 57% (G.3 ≥ 2%) ICANS 2% (G.3 ≥ 0%) Neutropenia G.3 ≥ 39.1%
EPCO-R^2^	Falchi, 2026 [95]	3	488	1 (1–2)	83/95	Not reached	CRS 35% (G.3 ≥ 0%) ICANS <1% (G.3 ≥ 0%) Neutropenia G.3 ≥ 69%

Acalabrutinib-R^2^, Acalabrutinib-Rituximab-Lenalidomide; AE, adverse events; Axicel, Axicabtagene; CRS, cytokine release syndrome; GADOLIN, GA101 (Obinutuzumab) and Dexamethasone; GALEN, GA101 (Obinutuzumab) and Lenalidomide; ICANS, immune effector cell-associated neurotoxicity syndrome; Lisocel, Lisocabtagene Maraleucel; Loncastuximab-R, Loncastuximab-Rituximab; mt, mutated-type; OB, Obinutuzumab; PolaBR, Polatuzumab Vedotin-Bendamustine-Rituximab; PolaBO, Polatuzumab Vedotin-Bendamustine-Obinutuzumab; R-B, Rituximab-Bendamustine; R-CHOP, Rituximab-Cyclophosphamide, Hydroxydaunorubicin, vincristine, Prednisone; R^2^, Rituximab-Lenalidomide; ROSEWOOD, (Zanubrutinib-O), Zanubrutinib-Obinutuzumab; Tazemetostat, EZH2 inhibitor; Tazemetostat-R^2^, EZH2 inhibitor-Rituximab-Lenalidomide; Tisacel, Tisagenlecleuce; wt, wild-type.
